# Are mucous retention cysts and pseudocysts in the maxillary sinus a risk factor for dental implants? A systematic review

**DOI:** 10.4317/medoral.24155

**Published:** 2020-11-28

**Authors:** Eduardo Anitua, Mohammad Hamdan Alkhraisat, Aintzane Torre, Asier Eguia

**Affiliations:** 1 University Institute for Regenerative Medicine and Oral Implantology – UIRMI (UPV/EHU-Fundación Eduardo Anitua), Vitoria, Spain; 2 BTI Biotechnology Institute, Vitoria, Spain; 3 Associate Professor. University of the Basque Country UPV/EHU. University Institute for Regenerative Medicine and Oral Implantology – UIRMI (UPV/EHU-Fundación Eduardo Anitua), Vitoria, Spain

## Abstract

**Background:**

Mucous retention cysts and pseudocysts of the maxillary sinus are benign lesions present in up to 13% of adult patients. Different surgical approaches for sinus lift and dental implant placement in the presence of these lesions have been proposed.

**Material and Methods:**

A systematic review was performed following the PRISMA statement recommendations to answer the PICO question: Does the aspiration or removal of mucous retention cysts/pseudocysts before or during sinus lifting and dental implant placing, affect the survival of the implants? The study was pre-registered in PROSPERO (CRD42020185528). Included articles quality was assessed using the “NIH quality assessment tool” and “The Newcastle-Ottawa scale”.

**Results:**

Previous literature in this field is scarce and with a low level of evidence. There are no randomized prospective studies. Only 19 studies were identified, being composed of 2 cohort studies and 17 case series/reports. These studies involved 182 patients with a previous history of mucous retention cyst or pseudocyst in 195 maxillary sinuses where 233 implants were placed. The mean age of the patients was 45.5 (range: 12-80 years); 122 (67%) were male patients and 60 (33%) were female patients. The mean follow-up of the patients was 17.6 (range: 4-90 months). Only two fail was reported. No differences were identified in relation to the surgical approach or in relation to the removal/aspiration of the sinus lesion (prior to or simultaneous to sinus grafting) or not.

**Conclusions:**

The level of evidence was grade 4 according to the CEBM and further studies are needed to confirm this observations, but with the available data, dental implants placement after sinus lift procedure in patients with mucous retention cysts and pseudocysts seems to be safe and present high survival regardless on the removal of the lesion or not.

** Key words:**Dental implants, maxillary sinus, sinus lift, mucous retention cyst, pseudocyst.

## Introduction

**Figure 1:**
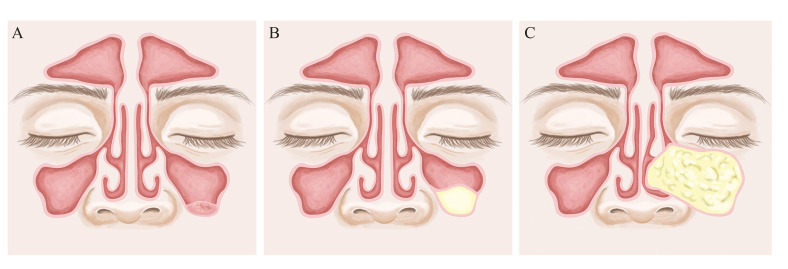
Differences between A- Pseudocyst, B- Mucous retention cyst and C-Mucocele.

Mucous retention cysts (MRC) and pseudocysts (PsC) of the maxillary sinuses, are benign, self-limiting lesions that originate from the accumulation of fluids inside the sinus membrane (1-3). MRC are the result of ductal obstruction of the seromucous glands (1,2). These “real cysts” possess a thin epithelial lining, while "pseudocysts" lack of an epithelial wall and originate due to diffuse subepithelial accumulation of inflammatory exudate (1-4). Both are radiologically indistinguishable, especially when they are not large lesions. Mucosal thickening over 2 mm. is defined by many authors as a pseudocyst, although there is no clear explanation about this definition (5). (Fig. 1).

It is really important to remark that in early literature about this topic, the term "pseudocyst" was often used to refer to both "cysts" and "pseudocysts". Probably, in order to differentiate them from the mucocele of the maxillary sinus, which has a different origin and lining and has a more expansive and aggressive growth that requires treatment. This fact can lead to misinterpretations when reviewing the literature (1,6).

MRC are incidental radiological findings in most cases and are seen in up to 13% of the adult population (7,8). They are usually asymptomatic, although occasionally they may produce headache, periorbital or facial pain and even exceptionally may predispose to the development of recurrent rhinosinusitis and produce nasal obstruction (1,2,6). In such uncommon cases, surgical treatment may become necessary. MRC are classically described as dome-shaped or rounded lesions originating in the maxillary sinus floor mucosa, although they may appear in other locations within the sinus. The size can be variable but the growth is normally slow. As time goes by in the absence of any treatment , in 60% of cases the size does not change, 30% decrease or even disappear and only 10% increase in volume (2).

The typical radiologic image is a dense, uniform, cupuliform or "rising sun" image, with well defined margins that perfectly respect the underlying bone structures (2,8,9). (Table 1) (Fig. 1). Except in cases where symptoms are present, they do not require specific treatment, but it is absolutely necessary to ensure a correct diagnosis in all cases (1-3). Computerized tomography is a critical tool for establishing the proper diagnosis (1-3). A differential diagnosis must be done with other benign but more aggressive pathologies such as the mucocele of the maxillary sinus, the nasosinus inverted papilloma and even with malignant pathologies such as the squamous cell carcinoma of the maxillary sinus (8,9). (Table 1, Table 2, Fig. 1).

**Table 1:**
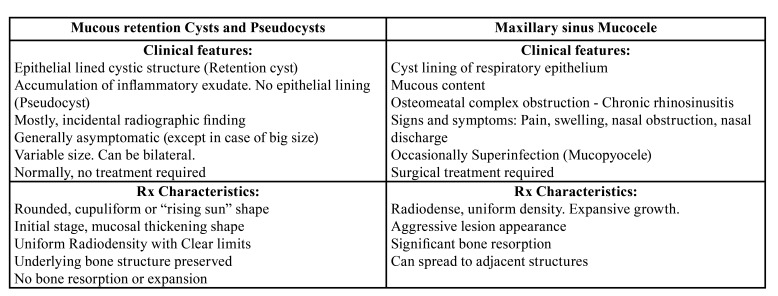
Summary of the main characteristics of MRC, PsC and Mucocele.

**Table 2:**
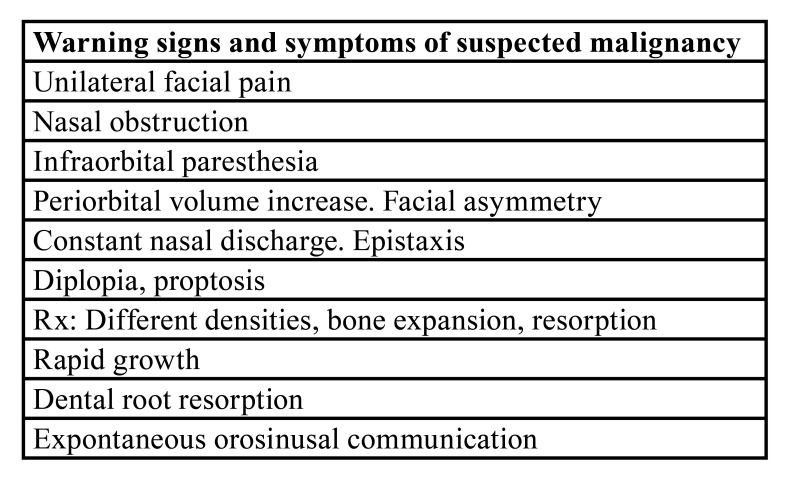
Malignancy suspicious signs and symptoms in a maxillary sinus lesion.

Various sinus lift techniques have proven reliable in the treatment of the atrophic posterior maxillary jaw (10,11,12). Both lateral and crestal approaches have been employed, being the remaining bone height the factor that can condition the choice for one or the other. There are several modifications in both original approaches such as the use of short and extra-short implants, that also provides good results (13,14). Time when dental implants have been placed (immediate or delayed after sinus grafting) has not been a factor that influences the survival of the implants (11-12,15).

Different approaches have been proposed in the literature to place implants in patients with MRC and PsC, when sinus floor elevation is needed (16-18). Initial approaches to these situations considered their presence as a contraindication and recommended a previous access to the sinus and lesion removal. After a period of at least six months, sinus grafting could be performed either using a lateral or a transcrestal technique. In this second stage, implants were placed at the time of the sinus grafting or were placed in a third stage, depending on the size and quality of the subantral remnant bone (16,17). Nevertheless, in different more recent case series, several patients have been treated without lesion removal, in a one-stage approach, sinus lifting and implant placing at the same or a two-stages approach, delaying the implants placing (18,19). Currently there is still a controversy whether the lesions should be removed / aspirated or not before sinus lifting and implant placement (4,20). This systematic review has been conducted in order to answer the following question: Are mucous retention cysts and pseudocysts in the maxillary sinus a risk factor for dental implants?

## Material and Methods

A systematic review was carried out following the Preferred Reporting Items for Systematic Reviews and Meta-Analyses (PRISMA) statement recommendations (21) in order to answer the following the PICO questions: Does the aspiration or removal of mucous retention cysts/pseudocysts before or during sinus lifting and dental implant placing, improve the success rate of the implants? and as a secondary question, Do people with previous mucous retention cyst/pseudocyst present lower implant survival after sinus lifting and dental implant placing?

- Protocol and registration:

A register in the International Prospective Register of Systematic Reviews - PROSPERO (NIHR) was obtained before starting (CRD42020185528). The PRISMA guide for systematic reviews was used to conduct the review process (21).

To build the search strategy, the following considerations were applied: Patient with a previous diagnosis of mucous retention cyst or pseudocyst who require sinus lift and dental implant placement, Intervention- sinus lesion removal before or during sinus lift and dental implant placement (in one stage or delayed), Comparison- patients in which sinus lesions have been untreated and radiologically controlled, Outcomes- Implant survival.

- Eligibility criteria, information sources and search

Data sources for this study were Medline, DOAJ and SCOPUS. The search was carried out using both medical subject heading (MeSH) and free terms. The search strategy applied was: ((mucous retention cyst) OR (pseudocyst)) and ((dental implants) OR (sinus lift) OR (sinus graft) OR (sinus floor augmentation)). Articles between January 1990 and May 2020 were initially selected. This range was defined based on prospecting previous searches and to avoid including very outdated information. Prospective, retrospective and cross-sectional studies, randomized and non-randomized, case reports and case series were included. Exclusion criteria were: narrative reviews, studies without a follow-up after implant placement or no previous diagnosis of mucous retention cyst/pseudocyst before implant placement, articles in other languages than English or Spanish.

- Study selection

The study selection was performed by two independent reviewers. and an additional reviewer acted in case of disagreement. After article selection based on the abstract and the patient selection criteria, both reviewers read the complete articles and determined whether they actually met the inclusion criteria for this review. Agreement in the selection process was calculated using Cohen’s kappa coefficient, with a k value of 0,88.

- Data collection process:

Data from all articles was collected in duplicate by both researchers (AE & AT) independently and then pooled in the same worksheet.

- Data synthesis

The following information was extracted from each selected study: authors, year of publication, number of patients, number of sinuses treated and number of implants, sex, age, follow-up period, previous lesion remove of the lesion or not, surgical approach for sinus lifting (lateral os transalveolar), one stage or delayed implant placement, type of grafting material, removal or aspiration of the sinusal lesion during the sinus lift, survival rate of the implants, membrane perforation rate, number of cases of sinusitis, implant failure, acute sinusitis and suture.

- Risk of Bias in individual studies

The methodological quality of the included studies was assessed using The National Institutes of Health - “NIH quality assessment tool” for case reports and series and “The Newcastle-Ottawa scale for assessing the quality of nonrandomised studies” for case-control and cohort studies (22). Although “NIH quality assessment tools” were initially thought to help reviewers, these tools have been broadly used in many recent systematic reviews to assess the study quality (22).The risk of bias was measured independently by two authors, and in cases of disagreement, a third author participated to solve it. In order to rate the articles, a follow-up period ≥ 24 months was considered as “adequate” to rate this item.

- Summary measures

All the variables were collected in a database and analysed with IBM SPSS statistics v. 20-0 (IBM Corp., Armonk - NY, USA). For the univariate description, we employed basic descriptive statistics. The Chi Square statistic was used for testing relationships between categorical variables.

## Results

- Study selection

**Figure 2:**
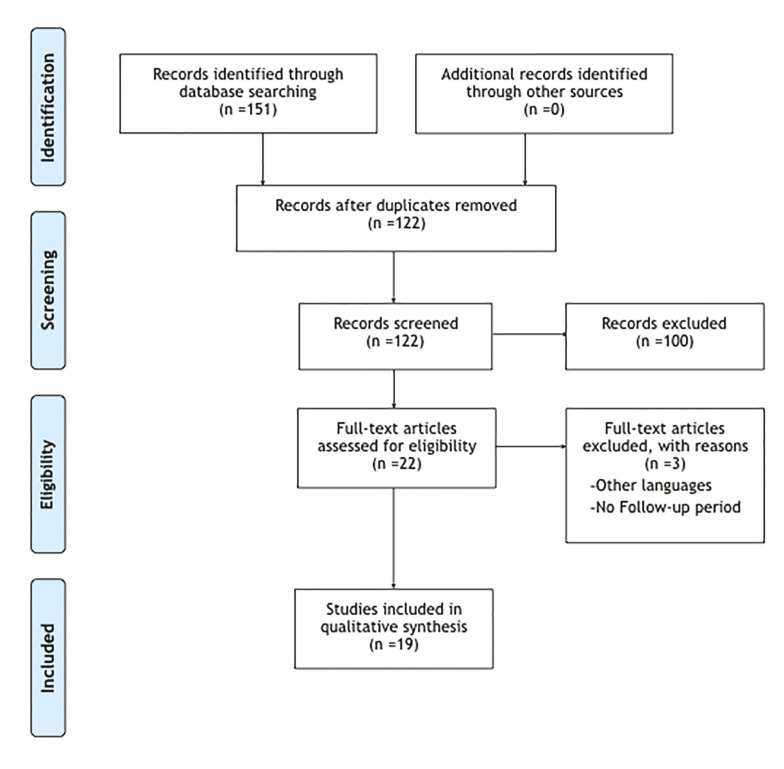
Article selection. Prisma Flow diagram.

The search strategy allowed us to initially identify 151 articles; 29 duplicated articles were eliminated, and after the screening of the other 122 articles, 100 articles were excluded because they were out of the focus of this review. After reading in detail the articles, three articles were additionally excluded due the absence of follow-up period or because the language was other than English or Spanish. Finally 19 articles were included for review (4,5,18-20,23-36). Fig. 2 summarizes the study selection process in a Flow Diagram.

- Study characteristics

The 19 articles involved a total of 182 patients (195 maxillary sinuses) in which a sinus lift was carried out, and 233 implants were placed. The mean age of the patients was 45.5 (range: 12-80 years), and 122 (67%) were male patients and 60 (33%) were female patients. The mean follow-up of the patients was 17.6 months [4-90]. The sinus grafting was performed through lateral access in 109 patients (60%), and a crestal access in 57 patients (31.4%). From the patients treated with a crestal approach, in 33 (18.5%) patients the use of osteotomes was specified, 1 (0.5%) patient was treated with endoscopic assistance and 2(1%) with a flapless computer guided surgery. In 16 patients (8.5%), the followed surgical technique was not specified. (Table 3).

One-stage approach (simultaneous grafting and implant placing) was performed in 121 (62%) patients and delayed (2-stage) approach in 47 (24%) cases. In 27 patients (14%), the implant surgery was not defined.

In most of the patients, 177 (91%), xenograft was selected as grafting material. Nevertheless, in 8 (4%) patients fresh frozen bone was employed, in 6 (3%) patients no grafting material was employed and in 4 (2%) the grafting material was not specified.

In 11 patients (6%), the sinus cyst/pseudocyst had been removed some months previous to the sinus lifting and implant placement. In 105 patients (54%) aspiration or removal of the cyst/pseudocyst was performed during the sinus grafting and in 62(31.5%) the lesion was left untreated. In 17 (9%) cases the treatment or not of the cyst/pseudocyst was not clearly stated.

The follow-up period ranged from 4 to 90 moths. Of 233, only two implant loss was reported, what means an overall survival of 99% of the implants regardless of the followed surgical approach. No statistical differences in the survival rate could be observed in relation to age, gender, surgical approach, implant surgery or grafting material.

No statistically significant difference in implant survival was observed in relation to the previous removal of the cyst/pseudocyst, the removal or aspiration during the sinus grafting or in the cases in which the lesion remained untreated.

Surgical complications reported were acute sinusitis and implant loss (1 patient), wound dehiscence (1) superficial abscess (1) and significant membrane perforation (2).

- Risk of bias across studies

Quality assessment was performed using the Newcastle-Ottawa tool in two cases, which corresponded to retrospective cohort studies, and in 17 studies using the National Heart, Lung, and Blood Institute's (NIH) Study Quality Assessment Tools for Case Series, since they were case reports or case series. Of the 19 articles, only one was rated as high quality (5.5%), 6 as medium quality (31.5%) and 12 as low quality (63%) (Table 4).

**Table 3:**
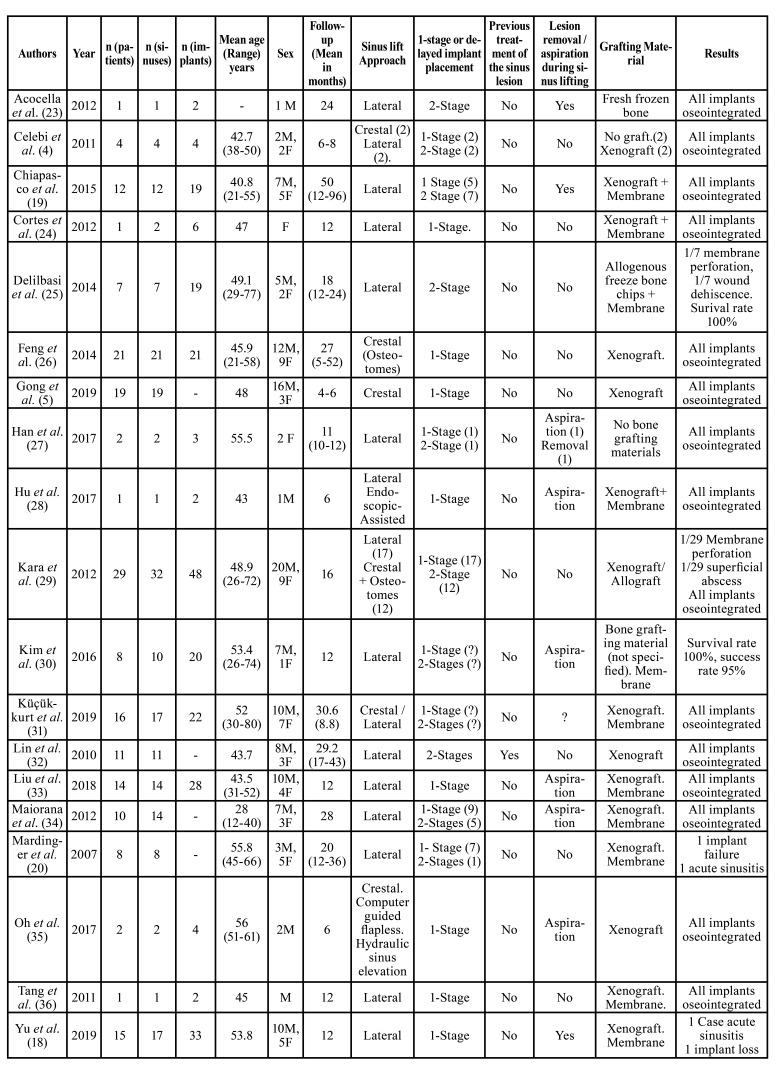
Patients data from the selected articles.

**Table 4:**
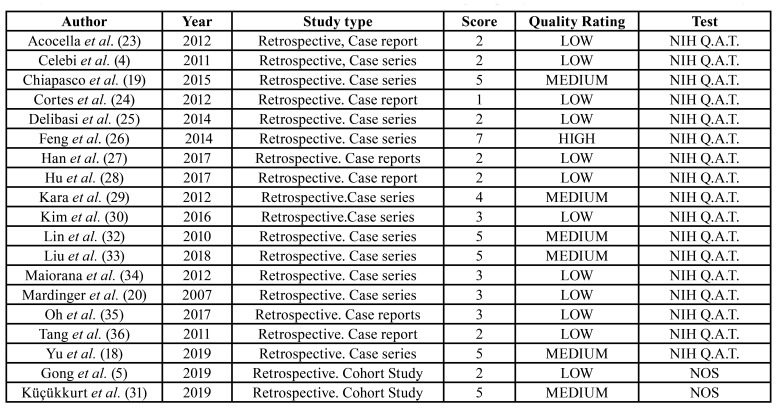
Quality assessment of the articles included in this systematic review. NIH QAT - National Heart, Lung, and Blood Institute's Study Quality Assessment Tools for Case Series. NOS - Newcastle Ottawa Test for assessing the quality of nonrandomised studies in meta-analyses.

## Discussion

The studies selected for this review showed a great heterogeneity of surgical approaches with similar results but with reduced numbers of patients and a low level of evidence. In patients with a previous history of MRC or PsC, no differences have been observed in the prognosis of the implants placed simultaneously or after sinus grafting regardless the surgical technique employed.

In the past, there were some controversies about the indication of sinus lift and implant placement in patients with MRC and PsC, without a previous removal of the lesion and a healing period (16,17) . However, different case series during the last years (4,20,25,26,32) have shown that lesions could be treated during the same surgical procedure of sinus lifting by aspiration or removal, without further complications. As can be seen in the results of this study, same implant survival and complications rates have been reported among patients with and without MRCs, when performing both sinus lift and implant placing regardless of the use of a one-stage or two-stage approach.

Despite this, many authors (18,30,34) recommend the aspiration and decompression of cysts during sinus lift surgery when possible. It has been stated that reduction of the size of the lesion by aspiration helps to decrease the internal pressure of the sinus decreasing too the risk of perforation of the sinus membrane. Despite all the controversies,in presence of symptomatic lesions or when there is an unclear diagnosis, enucleation should be considered (2,37).

The very low frequency of sinus membrane perforation and postoperative sinusitis and the published survival of implants suggest that maxillary sinus lift in patients with MRC and PsC are safe. However, as can be observed in this review, the studies are extremely heterogeneous and limited, so further studies are needed to confirm these observations.

Considering all the studies together, it is remarkable that after the follow-up period only two implant loss were reported, what meant an overall survival rate of 99% of the implants regardless of the followed surgical approach. It is possible that these results could be biased by several factors: the lack of randomization of cases, the fact that the authors did not include consecutive cases in many studies, and a possible unintended pre-selection of cases included for the study since all the studies were retrospective or without comprehensive inclusion criteria in many cases.

A very important confounding factor for this work is the fact that in many studies there is no clear consensus on the definitions of what a mucous retention cyst, a pseudocyst and a mucosal thickening exactly are, and what their differences are. For example, it is important to analyze in each study which is what each author understands by cyst or pseudocyst and whether they differentiate them or join them under the same name.

The absence of randomized clinical trials is a weakness. Moreover, in most papers, the inclusion and exclusion criteria were not clearly stated and only in very few studies, the case series were composed by consecutive cases. All this together with the previously mentioned heterogeneity of surgical approaches and the reduced numbers of patients, made it difficult to perform quantitative analysis. Other weaknesses were detected in the selected studies. Among others, important items were not specified such as the implant design or type of implants employed. All the papers report “survival rates” (defined as the maintenance of the osseointegration until the end of the follow-up period), but not treatment “success rates” (aesthetic outcome, peri-implant health maintenance or implant bone loss amongst others) (4,5,18-20,23-36).

Only in 7 studies (4,19,26,31,32,34) the follow-up is longer than 24 months (and not in all patients). It is therefore quite complicated to draw consistent conclusions in the medium to long term, both on the prognosis of the implants placed in these patients, and on the evolution of the sinus lesions. No precise information on the evolution of the sinus lesions after the follow-up period could be observed in most of the studies. However, no significant complications were reported in both treated (removal or aspiration) and untreated cases such as significant growth of the cyst/pseudocyst, onset of symptoms or recurrence of the lesion when treated.

Although no short-term complications have been reported, the medium to long term evolution of untreated MRC and PsC after sinus elevation and implant placement cannot be clarified due to the short follow-up periods and the small number of patients. New prospective studies with a long follow-up period would be desirable to clarify these old questions definitively.

## Conclusions

Conclusions of this review are based on few studies, usually underpowered, having short follow-ups, and often judged to be at high risk of bias, therefore they should be viewed as preliminary and interpreted with caution. The presence of a MRC or PSC has not been considered contraindication for sinus lifting and implant placement. Due to the short follow-up periods, the small number of patients and the lack of information in most studies, the medium to long term evolution of untreated MRC and PsC after sinus elevation and implant placing cannot be clarified. The removal or not of the lesions seem, not to affect the implant survival.
